# Association of acculturation and country of origin with self-reported hypertension and diabetes in a heterogeneous Hispanic population

**DOI:** 10.1186/1471-2458-12-768

**Published:** 2012-09-11

**Authors:** Fátima Rodriguez, LeRoi S Hicks, Lenny López

**Affiliations:** 1 Department of Medicine, Brigham and Women’s Hospital, 75 Francis Street, 02115, Boston, MA, USA; 2 Harvard Medical School, 25 Shattuck Street, 02115, Boston, MA, USA; 3 Division of Hospital Medicine, University of Massachusetts Medical Center and University of Massachusetts Medical School, 365 Plantation Street, Worcester, MA, USA; 4 Mongan Institute for Health Policy and Department of Medicine, Massachusetts General Hospital; Harvard Medical School, Boston, MA, USA; 5 Disparities Solutions Center, Massachusetts General Hospital, 50 Staniford Street, 02114, Boston, MA, USA; 6 The Division of General Internal Medicine and Brigham and Women’s Academic Hospitalist Program, Brigham and Women’s Hospital, 75 Francis Street, 02115, Boston, MA, USA

**Keywords:** Acculturation, Ethnicity, Hypertension, Diabetes, Hispanic

## Abstract

**Background:**

Hispanics are the fasting growing population in the U.S. and disproportionately suffer from chronic diseases such as hypertension and diabetes. Little is known about the complex interplay between acculturation and chronic disease prevalence in the growing and increasingly diverse Hispanic population. We explored the association between diabetes and hypertension prevalence among distinct U.S. Hispanic subgroups by country of origin and by degree of acculturation.

**Methods:**

We examined the adult participants in the 2001, 2003, 2005, and 2007 California Health Interview Survey (CHIS). Using weighted logistic regression stratified by nativity, we measured the association between country of origin and self-reported hypertension and diabetes adjusting for participants’ demographics, insurance status, socio-economic status and degree of acculturation measured by citizenship, English language proficiency and the number of years of residence in the U.S.

**Results:**

There were 33,633 self-identified Hispanics (foreign-born: 19,988; U.S.-born: 13,645). After multivariable adjustment, we found significant heterogeneity in self-reported hypertension and diabetes prevalence among Hispanic subgroups. Increasing years of U.S. residence was associated with increased disease prevalence. Among all foreign-born subgroups, only Mexicans reported lower odds of hypertension after adjustment for socioeconomic and acculturation factors. Both U.S.-born and foreign-born Mexicans had higher rates of diabetes as compared to non-Hispanic whites.

**Conclusions:**

We found significant heterogeneity among Hispanics in self-reported rates of hypertension and diabetes by acculturation and country of origin. Our findings highlight the importance of disaggregation of Hispanics by country of origin and acculturation factors whenever possible.

## Background

Hispanics are the fastest growing minority group in the U.S., comprising 15% of the population [1]. Compared with non-Hispanic whites, Hispanics have significantly higher rates of diabetes and hypertension [2-8]. These differences account for the disparity in cardiovascular-related morbidity and age adjusted mortality between Hispanics and non-Hispanic whites [9,10].

While disease prevalence has been shown to vary by Hispanic subgroups [11-14], few studies have examined the differential effect of acculturation across Hispanic subgroups defined by country of origin. Acculturation is defined as a multidimensional process through which foreigners adopt the customs of a host country [13]. While a standard acculturation metric is not fully agreed upon[15], language remains an important acculturation metric since more than three-quarters of the U.S. Hispanic population speak a language other than English at home [1]. Other important components of acculturation include: nativity (U.S.-born or foreign-born), years of residence in the United States and citizenship status [15]. Although Hispanics have been traditionally studied as a homogenous group [8], understanding determinants of chronic diseases among distinct subgroups remains salient in planning public health interventions.

Since the Hispanic population in California is two times greater than that of the remainder of the U.S. (36.2% versus 15.1%) [16], we examined the hypothesis that diabetes and hypertension prevalence among U.S. Hispanics varies by country of origin and by the degree of acculturation using the California Health Interview Survey (CHIS).

## Methods

We examined the adult participants in the 2001, 2003, 2005, and 2007 CHIS, the largest health survey conducted in any state. CHIS is a population-based random-digit dial telephone survey of California’s population conducted every other year since 2001. CHIS employs a multi-stage sample design and includes cellphone-only households. In addition, interviews are conducted in English, Spanish, and three Asian languages. Using CHIS allows sampling of non-Carribean Hispanics in larger numbers than what is possible through national datasets. A detailed description of the history, design, data collection, and available data for CHIS is available at the website [17].

### Variables

Patient demographic characteristics included age, sex, self-reported race [Hispanic, non-Hispanic White (NHW)], federal poverty level (FPL) (0-99%, 100-199%, 200-299%, ≥ = 300%), insurance (private, Medi-CAL, Medicare, uninsured), and education level (less than high school, high school/GED/vocational high school completion, some college or higher education). Self-identified Hispanics were categorized by country of origin: Mexican, Salvadorean, Guatemalan, Central American, South American, Puerto Rican, Hispanic European, Hispanics having two or more countries of origin (2+ Hispanic), and Other Hispanic. We merged Salvadorean, Guatemalan and Central American into one category to account for smaller sample size. Other Hispanic included those who self-identified as this category and those who identified as Hispanic European. We did not include Puerto Ricans in our analyses due to small sample size.

Given the multiple metrics for measuring acculturation [15], we examined four variables as measures of acculturation based on their utilization in prior studies [13,18]. Self-reported English language ability was dichotomized from a four level categorical variable into not well (not at all, not well) and well (well, very well). Years of residence in the U.S. was categorized as a four level variable (≤1 yr-4 yrs, 5–9 yrs, 10–14 yrs, 15+ yrs). Nativity was defined as U.S.- or foreign-born. Citizenship was defined as U.S. citizen, naturalized citizen, and no-citizenship status. Years of residence in the U.S. and citizenship questions were only asked of those who were foreign-born.

Self-reported hypertension and diabetes were ascertained by the questions: “Has a doctor ever told you that you have high blood pressure?” and “Other than during pregnancy, has a doctor ever told you that you have diabetes or sugar diabetes?” Body mass index (BMI) was calculated by CHIS based on self-reported participant’s weight and height. Smoking habits were assessed with two questions: “Altogether have you smoked at least 100 or more cigarettes in your entire lifetime?” and “Do you now smoke cigarettes every day, some days or not at all?” Based on examination of distribution plots, self-reported intensity of utilization of clinical services in the past 12 months was defined into three categories: no visits, 1–11 visits, greater than 11 visits.

### Statistical analyses

We used the chi-square test to compare the distribution of participants’ demographic and clinical characteristics of self-reported hypertension and diabetes. Weighted logistic regression models were created for participants’ self-reported hypertension and diabetes. All models were adjusted for participants’ demographic characteristics, insurance status, socioeconomic level, clinical characteristics, and acculturation factors (years in the U.S., citizenship, and English language ability). NHW was used as a reference group. Models were stratified by nativity status because prior work has shown that significant differences exist between foreign- and U.S.-born Hispanics [11]. Because the prevalence of hypertension and diabetes increases with age, we decided *a priori* to conduct separate sensitivity analyses stratifying the aforementioned regression models by age greater than 45 years. Finally, separate interaction analyses were conducted between Hispanic country of origin subgroups and two acculturation variables (years of residence in the U.S. and English language ability).

Due to the complex sampling design of CHIS, SUDAAN 9.2 statistical software (Research Triangle Park, North Carolina) was used for all analyses. Special sample weights provided by CHIS were included in the analysis to adjust for oversampling, non-response bias and post-stratification population totals. All p values reported are for 2-tailed tests and a value of <0.05 was considered statistically significant. All p values were adjusted for multiple comparisons between subgroups using the Bonferroni method.

The current study was deemed exempt by the Massachusetts General Hospital Institutional Review Board because the data were de-identified by CHIS and are publicly available.

## Results

There were 33,633 self-identified Hispanics and 126,448 NHWs. There were 13,645 U.S.-born Hispanics (Table 1) and 19,988 foreign-born Hispanics (Table 2). Total self-identified Hispanic subtypes included: Mexican (n = 26,091), Central American (n = 3,092), South American (n = 921), 2 + Hispanic (n = 2,245), and Other Hispanic (n = 1,284). Overall, regardless of nativity status, Hispanics were slightly younger than NHWs. Since our hypertension and diabetes findings did not change in separate sensitivity analyses stratifying for age greater than 45 years, our data is not presented stratified by age.

**Table 1:** Demographic Characteristics of US-Born Hispanic Adults by Subgroups

**US-Born**	**Mexican**	**Central American**	**South American**	**2+ Hispanic**	**Other Hispanic**	**Non-Hispanic White**
n	10, 433	333	162	1832	885	116, 122
Age, Mean (SD)	41 (17)	31 (13)	35 (13)	39 (16)	48 (17)	54 (17)
Female	50	43	52	52	46	51
Education						
< High School	4	2	0	3	4	2
High School/GED	56	50	30	51	44	33
College	40	48	70	45	51	65
Poverty Level						
0-99% FPL	16	19	9	16	10	5
100-199% FPL	23	24	11	21	19	12
200-299% FPL	17	14	12	17	18	13
≥300% FPL	43	44	67	46	53	69
Insurance						
Private	62	52	75	66	62	71
Medicare	6	1	1	5	8	16
Medicaid	16	22	8	11	16	6
Uninsured	17	26	16	18	14	7
Utilization						
Utilization of Care/year						
No visits	19	23	27	19	17	14
≥1 to <11 visits	73	73	68	74	74	76
≥11 visits	8	4	6	7	9	9
% Clinical						
BMI						
Normal	46	50	61	56	47	58
Overweight	28	26	30	25	31	27
Obese	26	24	9	19	22	15
Ever Smoke	34	27	25	32	42	47
Hypertension	21	6	11	19	25	27
Diabetes	9	1	0.50	7	13	7
% Acculturation						
Speak English Not Well or Not At All	5	5	0	2	1	1

All p values less than 0.05 compared to Non-Hispanic whites.

**Table 2:** Demographic Characteristics of Foreign-Born Hispanic Adults by Subgroups

**Foreign-Born**	**Mexican**	**Central American**	**South American**	**2+ Hispanic**	**Other Hispanic**	**Non-Hispanic White**
n	15, 658	2,759	759	413	399	10, 326
Age, Mean (SD)	40 (14)	42 (14)	46 (15)	44 (16)	52 (16)	53 (17)
% Demographics						
Female	48	52	57	44	51	52
Education						
< High School	45	39	6	19	16	6
High School/GED	42	39	32	41	32	26
College	13	21	62	40	52	68
Poverty Level						
0-99% FPL	40	35	19	23	17	8
100-199% FPL	36	36	25	29	21	14
200-299% FPL	12	12	17	16	13	13
≥300% FPL	12	17	39	32	49	65
Insurance						
Private	40	42	60	58	51	68
Medicare	2	2	6	5	17	15
Medicaid	20	19	14	16	14	8
Uninsured	38	37	21	21	19	9
Utilization of Care/year						
No visits	30	27	17	24	16	17
≥1 to <11 visits	65	68	77	70	78	75
≥11 visits	5	5	6	5	6	7
% Clinical						
BMI						
Normal	47	50	61	64	61	63
Overweight	32	28	27	22	23	26
Obese	21	22	12	15	16	11
Ever Smoke	30	25	39	39	37	46
Hypertension	16	19	17	20	37	23
Diabetes	9	9	5	8	10	6
% Acculturation						
Speak English Not Well or Not At All	72	61	37	31	26	13
Years in US^*^						
≤1 – 4	12	12	14	9	6	11
5 – 9	18	14	21	7	7	13
10 – 14	14	12	12	9	4	9
>15	56	62	54	75	82	68
Citizenship Status*						
Naturalized	28	34	51	58	70	66
Not a citizen	72	66	49	42	30	34

All p values less than 0.05 compared to Non-Hispanic whites.

*Years in US and citizenship status only asked to foreign-born adults.

### U.S.-born Hispanics

South American U.S.-Born Hispanics reported the highest rates of education and income level as compared with other Hispanic subgroups (Table 1). Mexicans, Central Americans, and Other Hispanics reported the highest rates of hypertension and obesity. Mexicans, 2+ Hispanics and Other Hispanics reported the highest rates of smoking among U.S.-born Hispanics. U.S.-born Hispanics rarely reported English language barriers.

In multivariable adjusted models, Mexicans, Central and South Americans were less likely to report hypertension as compared with NHWs (Table 3). Similarly, Mexicans and Other Hispanics were more likely to report diabetes while Central and South Americans were less likely to report diabetes as compared with NHWs.

**Table 3:** Multivariate Adjusted Odds Ratios of Self-Reported Hypertension and Diabetes among Hispanic Subgroups by Nativity

	**Self-Reported Hypertension**				**Self-Reported Diabetes**			
**Subgroup**	**U.S.-Born [OR (95% CI)]**	**P value**	**Foreign-Born [OR (95% CI)]**	**P value**	**U.S. Born [OR (95% CI)]**	**P value**	**Foreign-Born [OR (95% CI)]**	**P value**
**Mexico**	0.90 (0.77, 1.06)	0.59	0.83 (0.68, 1.01)	0.71	1.98 (1.58, 2.48)	<0.001	1.72 (1.31, 2.25)	<0.001
**Central America**	0.27 (0.12, 0.64)	<0.001	1.07 (0.86, 1.33)	0.93	0.37 (0.14, 1.01)	0.80	1.37 (1.10, 1.97)	0.02
**South America**	0.19 (0.08, 0.46)	<0.001	0.79 (0.55, 1.13)	0.58	0.32 (0.19, 0.51)	<0.001	1.59 (0.88, 2.87)	0.65
2+ Hispanic*	0.98 (0.70, 1.35)	0.90	1.53 (0.84, 2.80)	0.79	1.39 (0.90, 2.13)	0.42	0.72 (0.43, 1.19)	0.20
**Other Hispanic**	1.20 (0.74, 1.93)	0.49	1.78 (1.15, 2.73)	0.01	2.43 (1.42, 4.13)	<0.001	1.08 (0.47, 2.49)	0.57
**Non-Hispanic White**	**ref**	**ref**	**ref**	**ref**	**ref**	**ref**	**ref**	**ref**

*Participant reports ≥ 2 countries of origin. OR = Odds Ratio; CI = Confidence Interval. All models adjusted for age, gender, insurance status, education level, poverty level, healthcare utilization, diabetes, weight, smoking status, and acculturation metrics (self-report English language ability, citizenship, and years in the US).

### Foreign-born Hispanics

Foreign-born Hispanics reported a lower educational attainment level, as compared with their U.S.-born counterparts (Table 2). Mexicans and Central Americans were more likely to have the lowest incomes and to be uninsured compared with other foreign-born Hispanic subgroups. On the contrary, South Americans, 2+ Hispanics, Other Hispanics had the highest incomes and the highest rates of private insurance. Utilization of care rates (1–10 visits in the past year) was similar among groups regardless of nativity status. Mexicans and Central Americans had the highest rates of obesity across all Hispanics subgroups. South Americans, 2+ Hispanics and Other Hispanics had the highest rates of smoking. Foreign-born Mexicans and Central Americans were more likely to report not speaking English well compared with other Hispanic subtypes. Foreign-born Other Hispanics and 2+ Hispanics had the highest rates of greater than 15 years of residence in the U.S. and naturalization compared to the other Hispanic groups. The majority of the remaining Hispanic groups were not U.S. citizens.

Other Hispanics and 2+ Hispanics had the highest rate of hypertension among all groups and Mexicans had the lowest rate compared to NHWs (Table 2). In multivariable adjusted models, foreign-born Mexicans had the lowest odds of reporting hypertension and Other Hispanics the highest odds compared with NHWs (Table 3). Non-citizens, those with less than five years of residence in the U.S. and those with 10–14 years of residence in the U.S. were less likely to report hypertension in adjusted models (Table 4). After adjustment for clinical and demographic co-variates, limited English proficiency was not associated with self-reported hypertension.

**Table 4:** Effect of Acculturation On Self-Reported Rates of Hypertension and Diabetes Among Foreign Born Hispanics*

**Acculturation Variable**	**Hypertension**	**Diabetes**
**English Ability**	OR (95%CI)	OR (95%CI)
Not Well	1.17 (0.99 – 1.37)	1.07 (0.84 – 1.35)
Well	----	----
**Years in the US**		
≤1 – 4	0.75 (0.58 – 0.97)	0.45 (0.28 – 0.72)
5 – 9	1.00 (0.79 – 1.26)	0.57 (0.39 – 0.83)
10 – 14	0.79 (0.64 – 0.99)	0.71 (0.53-0.95)
>15	----	----
**Citizenship**		
Not citizen	0.84 (0.73 – 0.97)	1.18 (0.98 – 1.43)
Citizen	----	----

OR = Odds Ratio; CI = Confidence Interval.

*Acculturation variable odds ratios are from the fully adjusted models including demographic and clinical participant characteristics.

### U.S.-born versus foreign-born Hispanics

As compared with foreign-born Hispanics, U.S.-born Hispanics had higher educational attainment and income levels (Tables 1 and 2). Table 3 shows the differential effects of nativity on self-reported rates of hypertension and diabetes, after adjusting for demographic and clinical co-variates. Both U.S.-born and foreign-born Mexicans had lower odds of self-reporting hypertension and higher odds of reporting diabetes as compared with NHWs. U.S.-born Other Hispanics had the highest odds of self-reporting diabetes while foreign-born Other Hispanics had the highest odds of self-reporting hypertension.

## Discussion

Using a large state sample of Hispanics over several years, our study has several important findings on the association of acculturation and chronic disease prevalence. First, self-reported prevalence of hypertension and diabetes varied by country of origin. Second, stratifying by nativity status demonstrated significant differences in disease reporting by country of origin. Third, among all foreign-born subgroups, only Mexicans reported lower odds of hypertension after adjustment for socioeconomic and acculturation factors. Fourth, acculturation, as measured by years of residence in the U.S. and citizenship status, was an important predictor of hypertension and diabetes. This suggests that acculturation may differentially impact Hispanic subgroups. Our findings highlight the importance of disaggregation of Hispanics by country of origin and acculturation factors whenever possible.

Nativity had important differential effects. Compared with NHW, we found that U.S.-born Mexicans, Central Americans South Americans and foreign-born Mexicans had lower odds of reporting hypertension. The rate of hypertension increased after adjustment for socioeconomic status and acculturation factors in all groups except for South Americans. There was no difference in the reported odds of hypertension compared to NHWs in fully adjusted models for the other Hispanic subgroups. As people spend more time in the U.S., lifestyle changes and a consequent increase in BMI may significantly lead to a poorer cardiovascular profile [19-21]. Our study also found that U.S.-born Hispanics had higher BMIs compared to foreign-born Hispanics, regardless of country of origin. However, changes in clinical risk factors might differ in each Hispanic subgroup. Prior studies have shown that despite similar cardiac risk factors, Hispanic subgroups have varying degrees of subclinical CVD, including higher coronary artery calcium (CAC) scores and inflammation as measured by C-reactive protein [12,22-24]. This may be attributed to the genetic diversity in Hispanic subgroups [25]. The low prevalence of hypertension among Mexicans, despite acculturation, might be explained by the geographic proximity to their native land, which may play a stronger role in preservation of family ties and traditions despite length of residency in the U.S. and English language acquisition [26].

The “Hispanic Paradox” refers to the epidemiological finding that foreign-born Hispanics – largely Mexican – often fare better than their white counterparts on morbidity and mortality outcomes, despite lower levels of income, education, and worse health care access [27-29]. This difference has been attributed to a healthy migrant effect, healthier behaviors, and/or cultural traditions. The healthy migrant effect posits that healthier persons are more likely to migrate thus producing increased longevity and health in the emigrant population. The acculturation hypothesis states that Hispanic cultural orientation results in healthier behaviors that result in better health outcomes and is thus protective against the effects of lower socioeconomic status in the U.S. [29]. Prior studies of the healthy migrant effect have focused primarily on Mexicans with few studies focusing on Hispanic subgroups [30].

 Similar to prior studies, we also found lower odds of reported hypertension among foreign-born Mexicans even after adjustment for acculturation [31]. However, we found no significant difference in the odds of reported hypertension compared with NHWs after adjustment for socioeconomic and acculturation factors among all other foreign-born Hispanic subgroups. Our findings suggest that acculturation may differentially impact Hispanic subgroups and highlights the importance of disaggregation of Hispanics by country of origin and acculturation factors.

In agreement with existing literature, we found that reported diabetes rates varied with country of origin [6,7,32]. In our study, Mexicans, regardless of nativity status, have higher odds of reporting diabetes compared to NHWs, even after adjusting for BMI. In contrast, U.S.-born Central and South Americans had lower odds of reporting diabetes compared to NHWs. In addition, U.S.-born Other Hispanic and foreign-born Central Americans had higher odds of reporting diabetes. These findings might be attributed to genetic and behavioral differences, which make different subgroups more susceptible to diabetes and the metabolic syndrome and not hypertension. We found an increasing trend in the odd ratios for reporting diabetes as the years of residence in the U.S. increased (Table 4). This is consistent with prior studies that have shown an inverse relationship between increasing acculturation and a less healthy diet and increased rates of obesity [5,8,21,30,33]. Our findings show a complex relationship between greater acculturation and diabetes, likely because of the distinct Hispanic subgroups and numerous acculturation metrics available in our study.

We found higher odds of reporting hypertension among foreign-born Other Hispanics and higher rates of diabetes among U.S.-born Other Hispanics compared with NHWs. These differences may be due to a higher socioeconomic and acculturation status for this subgroup. Compared to other Hispanic subgroups, the Other Hispanics had high rates of college education, higher incomes, private insurance, and higher unadjusted rates of hypertension and diabetes. Among the foreign-born Hispanics, Other Hispanics were the most acculturated with 82% living in the U.S. greater than fifteen years and most speaking English well. The self-identification as “Other Hispanic” may reflect people who do not fit a particular pre-established Hispanic subgroup (i.e., European Hispanics or Brazilian) or people who identify as biracial or bi-ethnic (i.e., participant with only one Hispanic parent). The self-identity of Hispanics is complex and is influenced by the country of origin and nativity status [16]. Future studies should include bi-racial or bi-ethnic Hispanics as they constitute a poorly studied subgroup.

### Strengths and limitations

A major strength of our study is its large sample size of Hispanics over a seven-year period that used the same sampling and data collection methodology in a state with one of the largest Hispanic populations. The survey questions allowed for a more detailed analysis of self-identified Hispanics by country of origin and acculturation factors than has been available in previous studies. We were able to assess acculturation in four dimensions (i.e., English language ability, years of residence in the U.S., citizenship, and nativity status). In addition, California’s Hispanic demographic patterns allowed for a larger and more representative sample of Hispanics from Central and South America, groups traditionally underrepresented in other datasets. Importantly, our data allowed for statistical analyses that controlled for important confounders such as intensity of clinical services utilization.

Our study had several limitations. First, our data are cross-sectional and thus associations found are not proof of causality. Second, the self-reported nature of hypertension and diabetes may cause underestimation of disease rates, particularly because many Hispanics are uninsured and have less access to regular sources of healthcare. However, self-reported data for hypertension and diabetes have been shown to be highly correlated with physicians’ records [34-36]. Similarly, self-reported English language ability, one of the metrics of acculturation, was self-reported, consistent with current U.S. census definitions. We lacked significant data on Caribbean Hispanics and Black Hispanics, which may represent a large segment of the Hispanic population in other parts of the U.S. Finally, Hispanics who self-identified as “2+ Hispanic” and “Other Hispanic” represent a heterogeneous group from multiple countries of origin. However, these groups represent primarily U.S.-born Hispanics. Inclusion of these categories is important since it is reflective of the complex demographic self-identity of Hispanics that should be included in analyses of Hispanics.

## Conclusion

In conclusion, we found significant differences in rates of self-reported hypertension and diabetes by Hispanic subgroups, nativity, and acculturation. Our findings underscore the importance of exploring within-group differences and heterogeneity among Hispanics in the U.S., differences that may account for the mixed and paradoxical associations between acculturation and various health indicators. Finally, better standardized data collection strategies that incorporate Hispanic subgroups and acculturation factors will allow for improved tracking of health status among Hispanics, which may better inform public health interventions.

## Abbreviations

BMI: Body mass index; CHIS: California Health Interview Survey; CVD: Cardiovascular disease; NHW: Non-Hispanic white.

## Competing interests

The authors declare that they have no competing interests.

## Authors’ contributions

FR contributed to the data interpretation and manuscript preparation. LH contributed to the study design and conception a well as manuscript revisions. LL contributed to the conception and study design, data acquisition and interpretation, statistical analyses, and manuscript drafting. All authors read and approved the final manuscript.

## Pre-publication history

The pre-publication history for this paper can be accessed here:

http://www.biomedcentral.com/1471-2458/12/768/prepub
